# Ecotoxicological Effects of TiO_2_ P25 Nanoparticles Aqueous Suspensions on Zebrafish (*Danio rerio*) Eleutheroembryos

**DOI:** 10.3390/nano14040373

**Published:** 2024-02-17

**Authors:** Melissa I. Ortiz-Román, Ileska M. Casiano-Muñiz, Felix R. Román-Velázquez

**Affiliations:** Department of Chemistry, University of Puerto Rico, Mayaguez Campus, Mayaguez, PR 00681, USA; ileska.casiano@upr.edu

**Keywords:** TiO_2_ P25 NPs, zebrafish embryos, ecotoxicology, physical malformation, lethal concentration

## Abstract

Among nanoparticles (NPs), titanium dioxide is one of the most highly manufactured worldwide and widely used in multiple products for both industrial use and personal care products. This increases the probability of release into aquatic environments, potentially affecting these ecosystems. The present study aimed to evaluate TiO_2_ P25 NP toxicity in zebrafish embryos and eleutheroembryos by evaluating LC_50_, hatching rate, embryo development, and chemical analysis of the TiO_2_ concentration accumulated in eleutheroembryo tissues. Zebrafish embryos ~2 h post-fertilization (hpf) were exposed to 75, 100, 150, 200, and 250 mg/L TiO_2_ P25 NPs for 48 and 96 h. A total of 40–60 embryos were placed in each Petri dish for the respective treatments. Three replicates were used for each treatment group. Ti^4+^ concentrations were determined by inductively coupled plasma optical emission spectrometry (ICP-OES), and a conversion factor was used to calculate the TiO_2_ concentrations in the tissues. The highest calculated concentrations of TiO_2_ in zebrafish larvae were 1.0199 mg/L after 48 h and 1.2679 mg/L after 96 h of exposure. The toxicological results indicated that these NPs did not have a significant effect on the mortality and hatching of zebrafish embryos but did have an effect on their development. LC_20_ and LC_30_ were determined experimentally, and LC_50_ and LC_80_ were estimated using four different methods. Up to 11% of embryos also presented physical malformations. These effects can be detrimental to a species and affect ecosystems. Physical malformations were observed in all treatments, indicating teratogenic effects.

## 1. Introduction

Titanium dioxide (TiO_2_) has long been recognized for its utility in sunscreens due to its ability to absorb ultraviolet (UV) radiation. The rutile structure of TiO_2_ white pigment, named after the mineral form of TiO_2_, has been extensively employed in paints and plastics for its efficacy. However, beyond its conventional use, TiO_2_ possesses a remarkably high refractive index, allowing it to effectively scatter light. This characteristic renders TiO_2_ a valuable white pigment, widely utilized in paints for its exceptional covering power. Moreover, TiO_2_ has extensive applications as a food additive, primarily serving as a whitening and coloring agent in various food products. Its use in candies, chewing gum, yogurt, and other food items enhances their visual appeal [1]. Additionally, TiO_2_ functions as a stabilizer in certain food formulations, contributing to the maintenance of consistency and texture in products such as sauces and dairy items. Furthermore, it serves as an inactive ingredient in pharmaceuticals, where it acts as a coloring agent to improve the appearance of tablets and capsules [2]. Nonetheless, the unique properties of TiO_2_ nanoparticles (NPs), including surface structure, shape, aggregation, size, and composition, have raised safety concerns among researchers [3]. These nanoparticles find broad applications in diverse consumer products such as tires, fuel cells, and electronics [4,5]. Recent advancements have led to the production of transparent nanoparticles, approximately 50 nm in diameter, which are too small to scatter visible light. Despite their transparency, they retain the ability to absorb harmful short-wavelength UV radiation. Consequently, they are being incorporated into sunscreens to address this concern, although questions persist regarding their potential harm to the skin.

TiO_2_ NPs are among the most widely manufactured and used NPs in the world [6]. With significant worldwide reserves in excess of 600 million tons, the estimated annual production of Ti metal is 90,000 tons, while the annual production of titanium dioxide (TiO_2_) is approximately 4.3 million tons [7]. The annual production of TiO_2_ NPs grows continuously from 50,000 tons in 2009 to an expected 2.5 million tons in 2025 [8]. Larger TiO_2_ NPs are also used to impregnate fabrics and make clothing with built-in sun protection. Industries feel that the applications of TiO_2_ NPs may be safe, but there are issues to consider when using NPs in sunscreens and cosmetics, as little is known about their metabolism and absorption through the skin. In addition, TiO_2_ NPs produce free radicals on the surface of the skin in the presence of sunlight and moisture, although it is thought that these would not survive long enough to penetrate the skin, where they could cause damage to living cells [9]. The release of TiO_2_ nanomaterials into the environment (air, water, and soil) has been demonstrated qualitatively [5]; however, quantification of the amount released is difficult because nanoparticles are insoluble in water and precipitate. The FDA stipulated limitation (approved as a food additive since 1996) for sunscreens is that the TiO_2_ concentration must be less than 25% of the final product [1]. It is worth noting that while TiO_2_ is considered safe for consumption in small amounts by regulatory bodies like the FDA and the EFSA (European Food Safety Authority), there has been some debate and concern over the safety of TiO_2_ nanoparticles in food products, especially at higher concentrations [1,10,11]. With the wide prevalence of sunscreen use and the lack of distinction between TiO_2_ nanomaterials and larger-sized particles, people are exposed to these particles and are largely ignorant of their effects [1]. Therefore, given the global use and anthropogenic activities related to the use of this product [12], research evaluating the safety and toxicity of TiO_2_ NPs should be of greater interest. Because there is a high possibility that they will reach bodies of water, there is one more pollutant that can affect aquatic ecosystems.

To date, limited research has been conducted on the potential toxicity of TiO_2_ P25 NP_S_ and their environmental impact, especially at higher concentrations [1]. Zebrafish (*Danio rerio*) embryos allowed us to test the potential toxicological effects of exposure to NPs [13]. This species is a small tropical freshwater fish with considerable tolerance to a wide variety of husbandry conditions. It has been employed as an experimental species in various research areas because of its small size, high fecundity, optical clarity, low cost, and ease of maintenance [14]. This model is excellent for extrapolating results to humans, owing to its high genetic compatibility. Seventy percent of the genes that code for proteins in humans are related to genes found in zebrafish, and 84% of the genes associated with human diseases have homologs in this animal [15]. Humans and wildlife share common environments and food chains, and the physiological and molecular pathways altered in response to toxic chemicals are often highly conserved among vertebrates [16]. Gene programming and development during the early stages of vertebrate life are highly conserved. There are significant similarities in the morphology of all vertebrate embryos, and the vertebrate zebrafish embryo model is of great importance [17]. Similarly, the transplacental transfer of toxins from the maternal body to the embryo and fetus of mammals [18] and eggs (embryos) becomes the source of embryonic exposure to xenobiotics in aquatic environments [17]. NPs may enter an organism via different routes, with the potential risk of causing detrimental effects. No specific studies have been conducted on TiO_2_ zebrafish embryo exposure to TiO_2_ P25 NPs LC_50_ and the bioaccumulation capacity of the embryo (refer to Table 1).

The present study aimed to evaluate the toxicity of TiO_2_ P25 NPs in zebrafish embryos and eleutheroembryos by evaluating the lethal concentration of 50% of the population (LC_50_), hatching rate, embryo development, and chemical analysis of the concentration of TiO_2_ accumulated in the tissue. Zebrafish embryos were acutely exposed to different TiO_2_ P25 NP concentrations to test toxicity and chemical analysis using inductively coupled plasma optical emission spectroscopy (ICP-OES). The NPs were first characterized to validate their structure, size, and morphology. Another aim of this study is to characterize the TiO_2_ nanoparticles to compare our findings with those reported by other researchers. The main objective of this study was to provide a better understanding of the toxicological effects of TiO_2_ P25 NPs on living organisms in aquatic ecosystems at higher concentrations than those considered safe for humans [1].

## 2. Materials and Methods

### 2.1. Fish Maintenance and Breeding: Zebrafish

Mature zebrafish (*Danio rerio*), wild type obtained from Caribe Fisheries Inc., Lajas, Puerto Rico (fish farm), stayed housed on a ZebTEC Benchtop zebrafish housing system (Tecniplast) with mechanical and activated carbon filtration and sterilization (ultraviolet 40 W disinfection) at an automatized 12/12 h light/dark cycle. The water temperature was set to 27 °C ± 1 °C, and the room temperature was set to 24 °C ± 1 °C. Reverse osmosis/UV water, commercial sea salts (Instant Ocean^®^ Sea Salt, (St. Blacksburg, VA, USA), and NaHCO_3_ were added to maintain pH and conductivity values of 7.0 ± 0.5 and 1000 ± 100 μS, respectively. This water is referred to as embryo water. Properties such as pH, conductivity, system water temperature, room temperature, and system volume were checked and recorded daily. Adult fish were fed twice a day on a Zeigler Adult Zebrafish Diet. The animal protocols used in this study were evaluated and approved by the Institutional Animal Care Use Committee (IACUC) of the University of Puerto Rico at Mayagüez (Office of Laboratory Welfare assurance number D20-01098).

### 2.2. Reproduction Process

Sexually mature zebrafish were selected and placed in crossing tanks (transparent plastic fish tanks) with a mesh to prevent fish from eating their eggs. The female-to-male ratio was no more than 1:2. The crossing tanks were placed overnight on a rack. Embryos were collected in a glass Petri dish and rinsed with embryo water the following morning.

### 2.3. Preparation of Aqueous Suspensions (TiO_2_ P25 Nanoparticles)

Five concentrations (aqueous suspensions) were prepared for the treatments: 75, 100, 150, 200, and 250 mg/L of commercial TiO_2_ P25 NPs (Acros Organics, Thermo Scientific Chemicals, Waltham, MA). Concentrations were suspended with embryo water (without having embryos) (RO/UV water with pH 7.0 ± 0.5 and conductivity 1000 ± 100 μS) and placed in the ultrasonic (Bransonic^®^, model 3510R-MT) for 15 min. The diluent was embryo water to simulate the natural environment of zebrafish embryos as well as possible and to avoid other possible variables that could affect the embryos. These relatively high concentrations were chosen for their extensive industrial use, as described above. According to Kiser et al., the daily titanium (Ti) load from TiO_2_ NPs released into the environment is estimated to be around 4.2 (mg/person)/day [7]. This suggests a substantial concentration of this product in water bodies. The concentrations are correlated with the local population size. Additionally, these nanoparticles tend to aggregate and settle in the soil, potentially leading to high accumulation concentrations. With limited information on the interaction between TiO_2_ NPs, either in the free or aggregated states and sediments, it can be anticipated that they will bind to bed sediments, leading to uncertain toxicological implications [27].

### 2.4. Characterization of TiO_2_ P25 Nanoparticles

The X-ray diffraction (XRD) analysis was carried out for commercial TiO_2_ P25 NPs using a Siemens D5000 X-Ray diffractometer (PANalytical) equipped with monochromatic Cu-K^α^ radiation of wavelength (λ = 1.5418 Å) generated at 40 kV and 40 mA. Data were obtained within the range of 20–80°, 2θ range with a step size of 0.02, and a dwell time of 1 s. The diffraction patterns of both anatase and rutile TiO_2_ P25 powders were compared with the Joint Committee on Powder Diffraction Standards (JCPDS) database. Scherrer’s equation was used to calculate the crystal size. Additionally, hydrodynamic radius analysis was performed using three different solvents: methanol, DI water, and embryo water for size comparison. The hydrodynamic size and surface charge of the NPs were measured using a Malvern Zetasizer Nano-ZS90 system for a suspension with a TiO_2_ P25 NP concentration of 100 mg/L. For each sample, five measurements were taken in disposable polystyrene cuvettes with a 1 mL sample capacity. The measurement conditions were 25 °C, 120 s for the sample to acclimate, 1.330 for the dispersant (water), 0.8872 cP for viscosity, 78.5 for dispersant dielectric constant, 12 measurement runs, and 532 nm for the laser source. The Brownian motion of the NPs was examined using the Zetasizer system utilizing Dynamic Light Scattering (DLS), and their size was then calculated using well-known theories. Additionally, the zeta potential of the NPs was calculated based on Henry’s equation using software made available by the Malvern Instrument (Zetasizer Software 6.12). The UV–Vis absorption spectrum (Hach^®^ DR6000™) of the TiO_2_ P25 NPs was obtained from 250 to 600 nm.

### 2.5. Fish Embryo Acute Toxicity Test

The test method was based on OECD guideline 236 [28]. Fertilized embryos were collected and washed with embryo water. Eggs spawned by zebrafish are approximately 0.7 mm in diameter [29]. Embryos were examined for vitality using a stereomicroscope (AmScope SM-2TZ-LED) and were randomly distributed to the experimental groups. The experimental design consisted of six groups: one control group and five experimental groups. Embryos were transferred to glass Petri dishes with TiO_2_ P25 NP treatment solution. Sixty embryos were placed in each dish, and three replicates were evaluated per treatment. The control treatment contained only the embryo water without TiO_2_ NPs. Embryos were continuously exposed for 96 hpf at 28 ± 1 °C in Benchmark’s (Benchmark Scientific, Sayreville, NJ, USA) My Temp™ Mini Digital Incubators (H2200-H) in glass Petri dishes under a photoperiod of 12:12 h light/dark and were not fed during exposure. After 24, 48, 72, and 96 h, the appearance, mortality, development, and abnormal behavior were visually inspected and recorded using a stereomicroscope (AmScope SM-2TZ-LED) and Trinocular Compound Microscope (OMAX M837SL) with an 18.0 MP Digital camera (OMAX A35180U3). Dead embryos or eleutheroembryos were removed and discarded. After concluding the experiment, the eleutheroembryos were immersed in ice baths (0 °C) for 30 min to euthanize them. Subsequently, they were transferred to 1.5 mL centrifuge tubes and stored in a freezer at −20 °C for subsequent procedures. No other reagents were used to euthanize the embryos and eleutheroembryos, and subsequent analyses were not performed. Three rinses with RO water were performed to remove the adhering particulates before weighing, freezing, and removing as much water as possible. Triplicates were also performed for 48 h experiments. These results were used to calculate the LC_50_ values. In these groups, 40 zebrafish embryos were placed in Petri dishes, and the same procedure was performed.

### 2.6. Quantification of TiO_2_

The concentration of titanium was measured in embryos and larvae exposed to TiO_2_ NPs P25 for 48 and 96 h. Zebrafish eleutheroembryos were washed three times to ensure that they did not have particulates attached to the surface of their bodies and to quantify their absorption and weight before freezing. Acidic digestion was performed with HNO_3_ 70% to decompose TiO_2_ into Ti^4+^. The samples were then added to glass test tubes with 5 mL HNO_3_. Immediately thereafter, they were placed in a bath of water (70–80 °C) on a hot plate for 30 min. At the end of the digestion, the samples were left to rest at room temperature. Immediately after, samples were placed in labeled 25 mL flasks with deionized water and then transferred to centrifuge tubes to carry out the analysis. The concentration of Ti^4+^ was measured by inductively coupled plasma optical emission spectrometry (ICP-OES). A calibration curve was constructed from 0 to 100 mg/L (r = 0.9999). The Ti concentrations were converted to the TiO_2_ concentrations.

### 2.7. Statistical Analysis

All statistical analyses were performed using Minitab Statistical Software version 21.1 (64-bit). Data are expressed as means ± standard deviation (SD). Statistical significance between each treatment solution and the control was determined using one-way analysis of variance (ANOVA) with a Dunnett, Tukey, Fisher LSD Method for hatching rate, % of physical malformation, and quantification of TiO_2_ in larvae tissue. Significance was established at *p* < 0.05.

## 3. Results

### 3.1. Characterization of TiO_2_ P25 Nanoparticles

**Figure 1:**
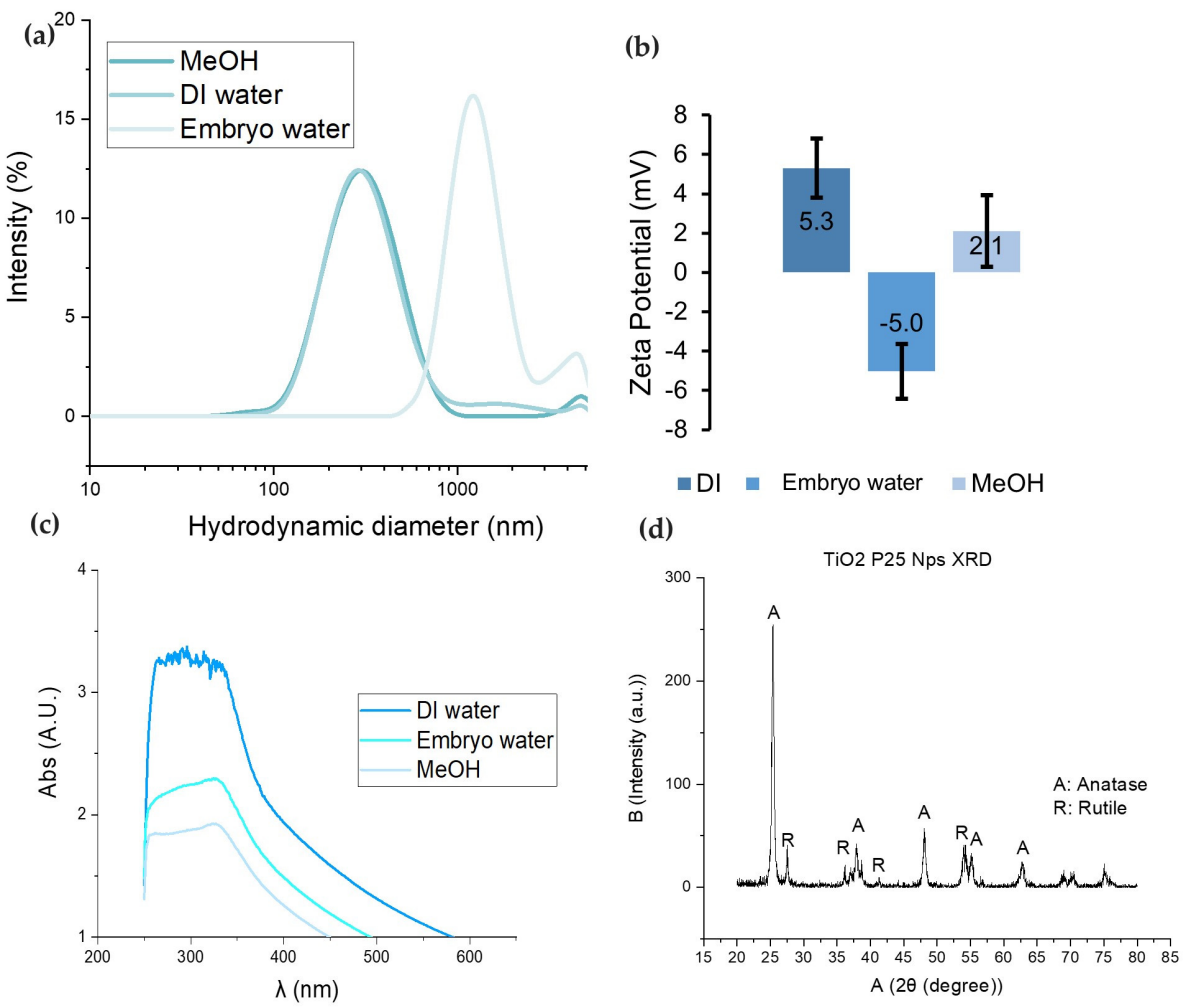
Characterization of TiO_2_ P25 NPs dispersed in various solvents: Zetasizer nano measurements of (**a**) hydrodynamic diameter, (**b**) zeta potential, and (**c**) UV–Vis absorption spectrum with standard deviations, (**d**) the XRD pattern shows five peaks at 25.378°, 37.900°, 48.061°, 55.123°, and 62.891° can be attributed to anatase; four peaks at 27.553°, 36.185°, 41.308°, and 53.958° can be attributed to rutile.

The X-ray diffraction analysis (XRD) pattern confirmed the presence of anatase and rutile components in the TiO_2_ P25 NPs. The characteristic peaks were compared with those reported by [30]. These peaks coincided well with those observed in the studied pattern. The sharpness of the peaks indicates that the TiO_2_ P25 NPs have a good crystalline nature. The strong diffraction peaks around 25.378° and 48.061° indicate the anatase phase, whereas the peaks around 27.553° and 53.958° indicate the rutile phase (Figure 1). The instrument obtained the size of the nanoparticles using the Scherrer equation. The average size of the TiO_2_ P25 NPs obtained was 50.4 ± 28.5 nm. Regarding the data obtained using the Zetasizer, an average hydrodynamic size of 2004 ± 137.4 nm was observed in the embryo water. This respective solvent presented a much larger size when compared to methanol (331.2 ± 1.8 nm) and DI water (357.5 ± 5.6 nm). The surface charge (Zeta potential) found for nanoparticles in suspension were -5.0 ± 1.4 mV in embryo water, 5.3 ± 1.51 mV in DI water, and 2.5± 1.82 mV in methanol, respectively (Figure 1). The spectra obtained in the wavelength range of 250–600 nm indicated a maximum wavelength of 327 nm for embryo water, 324 nm for methanol, and 296 nm for DI water. Using microscope images (40× magnification), it was possible to measure the size of the particle agglomerates, which had a maximum of 286.65 µm in diameter (Figure S1). 

### 3.2. LC_50_ Values of TiO_2_ P25 Nanoparticles and Hatching Rate for Zebrafish Eleutheroembryos

Reed–Muench [31], Probit [32], and Origin Pro v. 2018 (OriginLab Corp., Northampton, MA, USA, 2018) methods were used to calculate the lethal concentration. The LC_50_ value could not be determined experimentally at the concentrations used in this study but could be estimated. LC_20_, LC_30_, LC_50_, and LC_80_ were estimated using four methods (Table 2) to compare the precision of the results.

**Figure 2:**
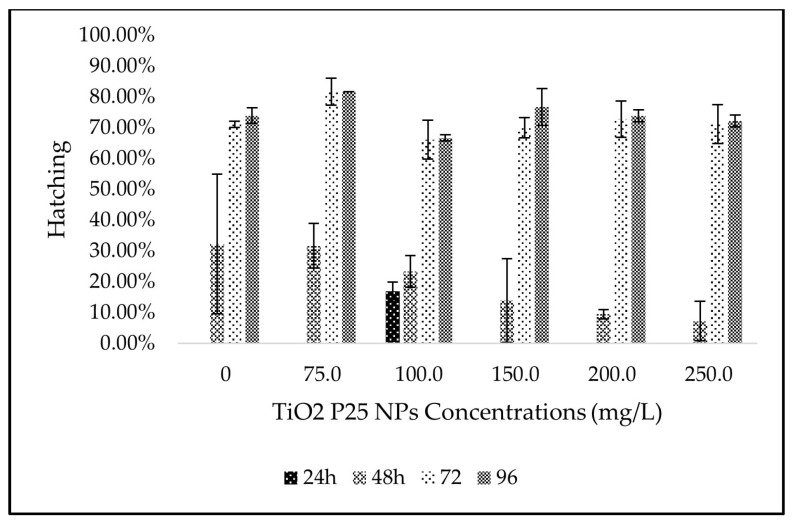
Average total hatching of embryos treated with TiO_2_ P25 NPs per group with standard deviations. No significant differences were identified between the groups at 24 h, 48 h, and 96 h. However, significant differences were noted at 72 h (*p* < 0.05). *n* = 3.

The hatching rate of zebrafish embryos exposed to TiO_2_ P25 NPs, with concentrations ranging from 75 to 250 mg/L for 48 h, is shown in Figure 2. Hatching rates were calculated cumulatively. Significant differences were only found in the % hatching groups at 72 h (*p* < 0.05) in the groups that were exposed for 96 h. The hatching rate at 48 h was higher in the control and 75 mg/L treatment groups. In the other experimental groups (above 100 mg/L), the hatching rate began to decrease as the concentration of TiO_2_ P25 NPs increased. Only in the 100 mg/L treatment did hatching occur at 24 h, which may indicate that it may cause early hatching.

### 3.3. Teratogenic Effects

**Figure 3:**
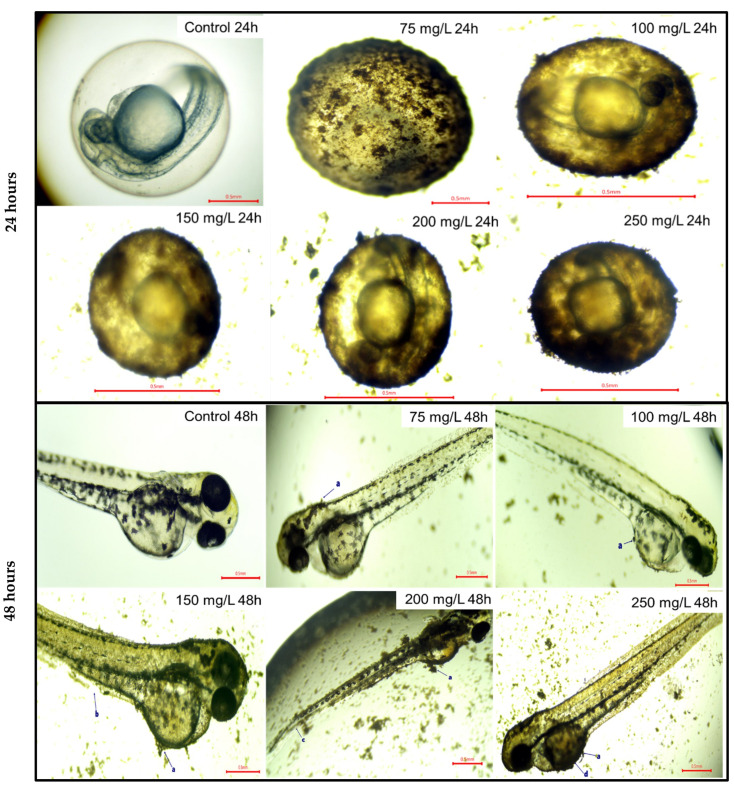
Zebrafish embryos at 24 and 48 h post-fertilization (hpf) following TiO_2_ P25 NP treatment. At 24 hpf, all treated embryos exhibit complete nanoparticle coverage. By 48 hpf post-treatment, adhered particulate matter and some instances of physical deformity become apparent. (a) adhered particulate, (b) tissue damage, (c) bent tail, and (d) opaque yolk.

**Figure 4:**
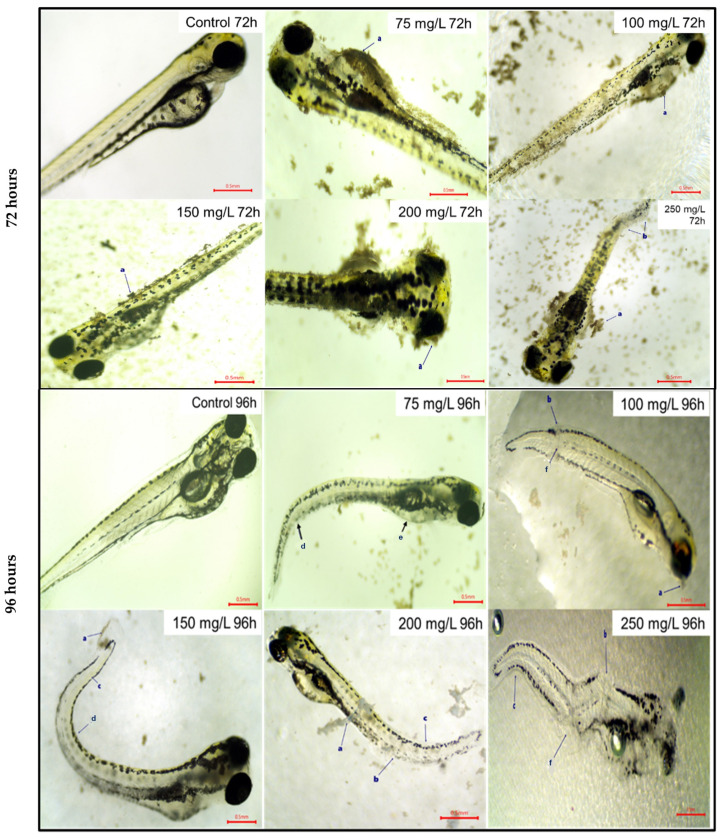
Zebrafish embryos at 72 and 96 h post-fertilization (hpf) following TiO_2_ P25 NP treatment. At 72 hpf, agglomerated particulate matter is observed in certain areas. (a) adhered particulate (b) tail folded in two parts (physical malformation). By 96 hpf, physical malformations were detected in all treatments except for the control. (a) adhered particulate (b) tissue damage (c) bent tail (d) scoliosis (e) uninflated swim bladder (f) split notochord.

**Figure 5:**
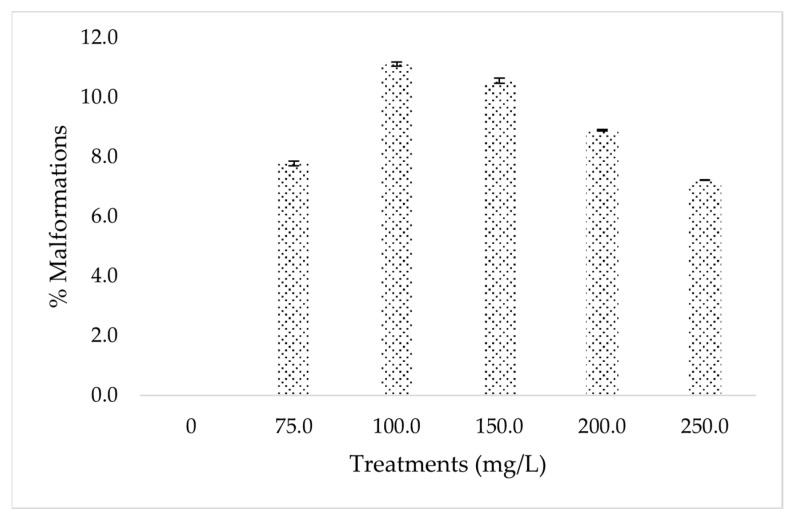
Physical malformations observed at 96 h of exposure with standard deviations. No physical malformations were identified in the control group. Significant differences were observed with the Fisher LSD method between the 100 mg/L and 150 mg/L groups compared to the control. *n* = 3.

The physical malformation rate was calculated with the data obtained at the end of the experiment at 96 h of exposure since, at this point, the physical development of the larva could be observed. In addition, we observed that TiO_2_ P25 NPs induced physical deformities during embryonic development. Among the physical malformations found in larvae were curved spines, growth retardation, bent tail, uninflated swim bladder, tissue damage, scoliosis, and larvae that never hatched but were alive (these looked like a shapeless mass of tissue, but the heart was beating) (Figure 3 and Figure 4). The groups that presented a higher percentage of physical malformations were at concentrations of 100 and 150 mg/L, with 14% malformation (Figure 5). No deformations were observed in the control group. All groups exposed to TiO_2_ P25 NPs presented some physical malformations. The group that presented the lowest percentage of physical deformation was the 75 mg/L group, with 7.8% malformation. The physical malformations that were mostly observed in the groups exposed to TiO_2_ P25 NPs had bent-tail problems and curved spines.

### 3.4. Quantification of Ti^4+^ Concentration in Zebrafish Eleutheroembryos

**Figure 6:**
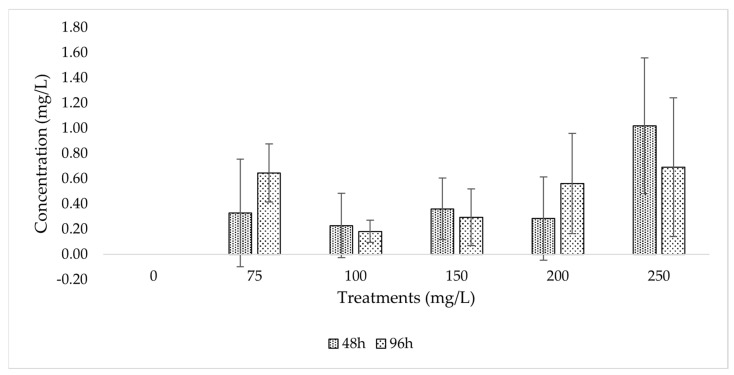
TiO_2_ concentration in zebrafish eleutheroembryos at 48 h and 96 h of exposure with standard deviations. Variations were observed among the groups at 48 h of exposure (*p* < 0.1, α = 0.1). At 96 h, significant differences were detected with the Fisher LSD method, specifically between the 250 mg/L group and all other groups. Tukey’s pairwise comparisons and Dunnett’s multiple comparisons with the control method revealed significant differences between the control group and the 75 mg/L, 200 mg/L, and 250 mg/L groups. *n* = 3.

To validate the method, five spikes were performed for each analysis, and a total of 10 spikes were performed, with a recovery percentage ranging from 93% to 114%. The limit of quantification was 0.007 mg/L, and the limit of detection was 0.002 mg/L. No Ti^4+^ concentration was found in the control groups at either exposure time. In all groups, there were different numbers of eleutheroembryos due to deaths associated with exposure. The wet weights of the samples exposed for 48 h and 96 h were in the range of 0.0113–0.0657 g and 0.0256–0.0745 g, respectively. The highest Ti^4+^ concentration was found in the group exposed to 250 mg/L of TiO_2_ NPs P25 at 48 h with 0.1223 mg/L of Ti^4+^ (1.020 mg/L TiO_2_) and at 96 h it was 0.0829 mg/L of Ti^4+^ (0.692 mg/L TiO_2_) (Figure 6). The Ti^4+^ concentrations found at 48 h among the groups exposed to TiO_2_ P25 NPs oscillate between 0.0292 and 0.1223 mg/L and at 96 h, they oscillate between 0.0219 and 0.0829 mg/L. The TiO_2_ concentrations calculated by the conversion factor at 48 h among the exposed groups ranged between 0.329 and 1.020 mg/L and at 96 h between 0.183 and 0.692 mg/L. The group with the lowest TiO_2_ concentration was the group exposed to 100 mg/L mg/L TiO_2_ NPs at both exposure times.

## 4. Discussion

### 4.1. Characterization of TiO_2_ P25 Nanoparticles

Numerous investigations have explored the toxicity of TiO_2_ NPs in various organisms employed as scientific models, including (TiO_2_ (99.0% pure), pure anatase TiO_2_ NPs, rutile polymorph, Nano-TiO_2_ (synthesized), and TiO_2_ P25, with other experiment-specific variables. This study specifically concentrated on commercially acquired TiO_2_ P25 NPs. Through techniques such as XRD, Zeta sizer, UV–Vis absorption spectrophotometry, and microscope imagery (40×), the evaluation encompassed composition, size, hydrodynamic size, surface charge, UV–Vis absorption spectrum, and particle agglomeration. Notably, a substantial difference in nanoparticle sizes was observed, ranging from 17 nm to 100 nm based on XRD results, indicating a lack of size homogeneity. However, the product specifications of the TiO_2_ P25 NPs indicated that the main size of this product was 21 nm. In contrast to a prior report [20,33], which indicated a primary particle size of 25 nm for TiO_2_ P25 NPs, composed of 20% rutile and 80% anatase, our study revealed an average size of the main particulate as 50.4 nm.

The substantial hydrodynamic size of TiO_2_ P25 NPs, measuring 2004.0 nm (±137.4), suggests their instability in embryo water compared to deionized water and methanol, commonly used as solvents. A prior study addressing the hydrodynamic diameter of TiO_2_ NPs in seawater, utilizing an Artemia medium, reported increased values ranging from 280 to 2334 nm [24]. In contrast, a study measuring 100 mg/L suspensions of TiO_2_ P25 NPs in the embryo exposure medium reported a hydrodynamic size of 1224.6 nm (±166.1) at 0 h [20], differing by over 700 nm from our study’s TiO_2_ P25 NPs in embryo water. This aligns with our findings, emphasizing nanoparticle deposition and aggregation, validating our study [34]. Li proposed that natural water could either stabilize or destabilize TiO_2_ NPs concerning aggregation and deposition, particularly in nutrient-rich lakes with elevated pH, electrical conductivity, alkalinity, and turbidity values [34]. Similar effects could manifest in river water, characterized by diverse chemical and physical properties in different bodies. Throughout our treatment solutions, the pH was maintained between 6.7 and 7.4, and conductivity ranged from 721 to 875 µS, aligning with the standard parameters of freshwater habituated by zebrafish.

The precipitation-inducing aggregation of nanoparticles in embryo water, which remained stable in deionized water and methanol, suggests a role played by salts in embryo water. A study noted that particles in aqueous solution displayed larger aggregate sizes in saltwater on the third day, indicating different behaviors of TiO_2_ NPs in saltwater compared to distilled water. The authors discussed the importance of adjusting pH to enhance surface charge or reducing ionic strength for increased colloidal stability [34]. Previous studies underscored the instability of these nanoparticles in natural waters. Notably, TiO_2_ NPs smaller than 20 nm may pose fewer environmental and ecotoxicological concerns due to more favorable conditions for aggregation, forming aggregates within minutes in aquatic environments ranging from hundreds of nanometers to several micrometers in diameter [25].

Ref. [5] argued that the high biological activity of TiO_2_ NPs, caused by their large specific surface area, creates a high potential for inflammatory, pro-oxidant, and antioxidant activity. Experimental findings indicate significant aggregation across all pH and ionic strength conditions, except for the lowest ionic strength observed at 0.0045 M NaCl and pH 4.5 [35]. In embryonic water, the zeta potential of suspended nanoparticles was negative, implying a positive charge similar to that observed in deionized water. Despite their positive charge, when present in a salted medium, these ions interacted with the nanoparticles through electrostatic forces, forming an external layer with a negative charge. Similarly, ref. [26] reported a negative zeta potential (−20.2 mV) of the NM-105 (P25) TiO_2_ in egg water suspension, aligning with our studies but with a distinct value. Notably, our research refrained from using stabilizers to mimic the natural aquatic environment better. It is evident that TiO_2_ P25 NPs lack stability in aquatic ecosystems, leading to their precipitation in the benthic environment where various organisms reside, reproduce, feed, and lay their eggs. This phenomenon has a discernible impact on the affected community.

### 4.2. LC_50_ Values of TiO_2_ P25 Nanoparticles and Hatching Rate for Zebrafish Eleutheroembryos

To assess lethal concentration values in zebrafish embryos, the Reed–Muench method was compared with Probit and Origin Pro results. Despite the absence of an identifiable LC_50_ under the studied conditions and concentrations, its estimation was possible using the exponential trendline equation by extrapolating graph data with the Reed–Muench method. LC_20_, LC_30_, and LC_50_ were estimated using four different methods for comparative analysis, revealing that an increase in the lethal concentration percentage corresponds to greater variability among values, leading to diminished precision. Upon scrutiny of LC_20_ and LC_30_ data, values exhibit proximity despite the use of distinct methodologies. In contrast, for LC_50_ and LC_80_, precision is notably lacking, with values displaying greater dispersion across the various methods. Consequently, it is asserted that, for the estimation of LC20 or LC_30_ values, any of the four utilized methods can be applied interchangeably. However, for LC_50_ or higher concentrations, the method involving graph estimation and calculation via the exponential equation appears to yield results with diminished variability.

Ref. [26] similarly failed to report LC_50_ at the studied concentrations (0, 0.01, 10, and 1000 mg/mL) but did report EC_10_ (0.073 mg/mL) and EC_50_ (107.2 mg/mL) for zebrafish embryos. The reported LC_50_ at 96 h by [12] for adult zebrafish exposed to TiO_2_ NPs (99% anatase), with a hydrodynamic diameter of 251–630 nm, was 124.5 mg/L. The LC_50_ for *Artemia salina* was reported as >100 mg/L [24,33]. For early Artemia sp. nauplii, the LC_50_ found at 48 h was 18.94 mg/L [25]. The estimated LC_50_ > 250 mg/L for zebrafish embryos after 24 h of exposure to TiO_2_ P25 NPs coincides with [20,24], indicating an LC_50_ is >100 mg/L. This article suggests that these nanoparticles might not significantly affect zebrafish embryo mortality within a 48 h exposure under the studied conditions, but they could significantly impact their development. Findings indicate that exposure to concentrations exceeding 200 mg/L could be detrimental to this fish population. Furthermore, results suggest that 220 mg/L TiO_2_ P25 NPs can induce a 30% mortality rate in an aquatic ecosystem population, potentially altering the food chain and ecosystem dynamics over the long term. The deposition of these nanoparticles in isolated aquatic ecosystems with minimal water flow at the bottom may lead to elevated concentrations.

Small-sized fish species can affect their population size at higher and lower trophic levels because of their mid-trophic position within the food web [36].

Cumulative hatching rates were computed, revealing significant differences solely in the % hatching groups at 72 h (*p* < 0.05). After 72 h of exposure, all groups presented approximately 70% hatched embryos. The hatching rate at 48 h was higher in the control and 75 mg/L groups and began to diminish as the concentration of TiO_2_ P25 NPs increased. As noted by [25], the presence of synthesized TiO_2_ NPs led to decreased hatching in *A. salina.* Interestingly, in our study, hatching occurred only in the 100 mg/L treatment at 24 h, suggesting a potential for early hatching. In contrast, [26] observed that NM-105 TiO_2_ NPs induced premature hatching and an overall reduction in the time required for normal hatching. However, the hatching rate of zebrafish embryos exposed to TiO_2_ NPs (99.5% anatase) suspensions showed no significant difference from the control, indicating the non-toxic nature of these suspensions to zebrafish embryo hatching under the given experimental conditions [37]. Notably, in our study, no relationship was identified between the concentration of exposure to zebrafish embryos and their hatching.

### 4.3. Teratogenic Effects

The current observations suggest the potential induction of somatic mutations by nanoparticles. Despite numerous studies on the exposure of aquatic organisms to TiO_2_ P25 NPs, no evidence of teratogenic effects has been identified. Zebrafish embryos exposed to TiO_2_ NP suspensions at various concentrations did not exhibit significant morphological abnormalities [38]. Wildlife populations exposed to environmental contaminants show clear evidence of congenital defects and reproductive anomalies [16].

Our findings reveal that up to 11.2% of the larvae exhibited physical malformations. While no direct correlation between exposure concentrations and physical deformations was observed, all exposed groups manifested some malformations. Conditions leading to skeletal deformities could be a crucial yet easily overlooked phenomenon, contributing to recruitment failure in contaminated aquatic habitats [39]. Physical malformations were present in all experimental groups, while the control group exhibited no observable malformations. Individuals exposed to environmental contaminants are likely more susceptible to predation or competition for food [39]. A developing organism is generally more vulnerable to environmental changes that can hinder its development. Most teratogens typically exert their effects during specific crucial stages of development [40]. Although exposure to TiO_2_ P25 NPs did not result in high mortality or a high % of physical malformations, individually, the combination of these outcomes for the same species may contribute to a population decrease in its natural habitat.

**Scheme 1:**
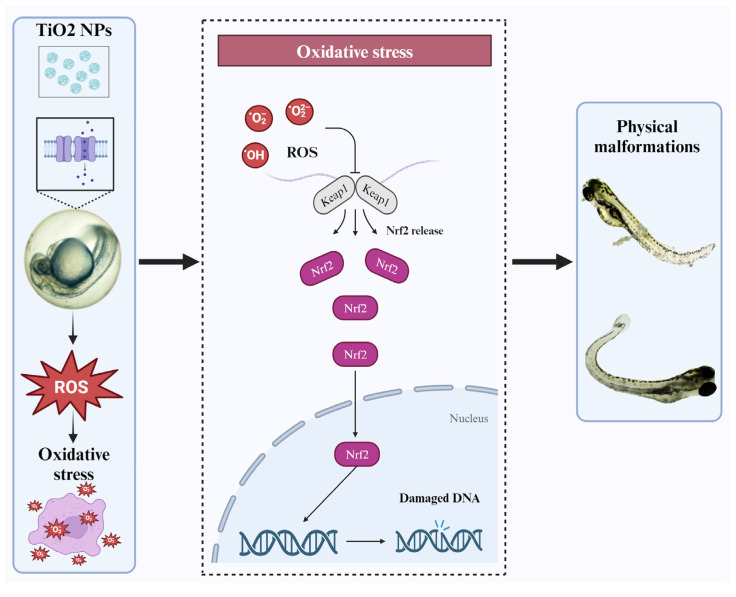
Suggested toxic mechanism of TiO_2_ NPs on zebrafish embryo.

One potential explanation for the observed results is illustrated in Scheme 1. TiO_2_ NPs have a greater capacity to induce the formation of reactive oxygen species (ROS) [41], leading to oxidative stress in cells [8,42,43]. Aggregates of TiO_2_ NPs have been shown to induce oxidative stress in zebrafish embryos [20,21]. This oxidative stress during embryonic development may result in abnormalities [44]. Oxidative stress caused by ROS increased abnormalities in the embryonic period. Changes in ROS and antioxidant levels can affect embryo development and physiological functions [45]. Nuclear factor-E2-related factor-2 (Nrf2) is the primary cellular defense against the cytotoxic effects of oxidative stress [44]. A potential mechanism involves Nrf-2 activation by TiO_2_ NPs, leading to reduced antioxidant activity [45] and DNA damage, which could contribute to the observed physical malformations in this study. When activated, Nrf-2 cleaves from Kelch-like ECH-associated protein 1 (Keap1), binds to the antioxidant response element, and stimulates phase II detoxification genes in response to oxidative stress. Keap1 functions as a negative regulator and promotes the rapid degradation of Nrf2 [46]. Previous research has linked malformed mice fetuses with ROS generation [47]. Considering the findings reported by previous studies, the morphological alterations observed in zebrafish embryos in this investigation could be attributed to the oxidative stress induced by nanoparticles.

### 4.4. Quantification of Ti^4+^ Concentration in Zebrafish Eleutheroembryos

The highest recorded TiO_2_ concentrations in zebrafish larvae were 1.0199 mg/L at 48 h and 1.2679 mg/L at 96 h post-exposure, which were relatively low compared to the exposure levels. However, the pore size of zebrafish chorion canals (approximately 600 to 700 nm) is smaller than the hydrodynamic radius of nanoparticles in embryonic water (2004.0 ± 137.4 nm), suggesting potential impacts on oxygen transport and nutrient availability, affecting embryo development. From fertilization to mouth opening, the embryo and preferred larvae of oviparous fish go through an initial endotrophic stage, in which they use endogenous nutrients from the yolk that have accumulated in the egg [48]. The nanoparticles can also agglomerate or interact with proteins on the surface of the chorion. The embryonic membrane contains several hydrophilic groups, such as carboxyl, amino, sulfate, phosphate, amide, and hydroxyimidazole [49], which can have electrostatic interactions with the surface charge of the nanoparticles. After 24 h of exposure, all embryos were covered with nanoparticles in all treatments. The interaction between nanoparticles and chorion proteins, along with the deterioration of the yolk sac, could hinder nutrient supply, impacting cardiac function due to energy limitations [50]. While nanoparticles could penetrate the thin skin of embryos (less than 10 µm thick) [51], interrupting development, natural aquatic environments may mitigate these effects. If we compare the size of the nanoparticles not agglomerated (50.4 ± 28.5 nm), they would have the ability to pass through the chorionic channels and could interrupt the development of the embryo. However, this is not what is expected to occur in natural aquatic environments.

## 5. Conclusions

This study is necessary to identify possible effects at the population level in zebrafish that can also be applied to other oviparous aquatic species; approximately 90% of bony and 43% of cartilaginous fish are oviparous [52] when exposed to TiO_2_ NPs. This chemical exposure could put species and reproduction rates at risk, especially if the keystone species were exposed. We attempted to imitate the natural aquatic environment without altering the ability of the nanoparticles to agglomerate in the presence of ions (other studies used anti-agglomeration agents). The results indicated that these nanoparticles did not have a great effect on the mortality and hatching of zebrafish embryos when exposed for 48 and 96 h under the conditions studied, but they did have a great effect on their development. LC_20_ and LC_30_ were found in up to 11% of physical malformations, consistent with the results obtained experimentally. This indicates a mortality rate of 30% of the population exposed to these nanoparticles and another 11% of the population who were more susceptible to dying. These numbers can be detrimental to a species and can affect population levels. Physical malformations were found at all concentrations, indicating teratogenic effects that can be attributed to oxidative stress induced by the nanoparticles. Further research is necessary to identify these effects in zebrafish embryos to simulate their natural environments. In addition, we suggest studying whether TiO_2_ P25 NPs affect the transport of oxygen and nutrients in the egg and, consequently, how embryonic development is managed.

## Figures and Tables

**Table 1:** Previous studies regarding the utilization of TiO_2_ P25 NPs.

Organism	Title/Authors	Material and Concentrations	Conclusions
Zebrafish embryos	Effect of titanium dioxide nanoparticles on zebrafish embryos and developing retina [19]	Commercial TiO_2_ NPs (P-25 type, 21 nm average size) 20, 10, 5, 1, 0.75, 0.5, and 0.2 mg/L	➢ “Short-term exposure to TiO_2_ NPs at a low dose does not lead to delayed embryonic development or retinal neurotoxicity”.
Zebrafish Embryos	Toxicity assessment of TiO_2_ nanoparticles in zebrafish embryos under different exposure conditions [20]	Commercial TiO_2_ NPs (P-25 type, 25 nm average size) 1, 10, and 100 mg/L	➢ Promoted a quicker hatching of the larvae. ➢ Changes in the activities of CAT and GST suggested the presence of oxidative stress.
Zebrafish embryos	Oxidative stress effects of titanium dioxide nanoparticle aggregates in zebrafish embryos [21]	TiO_2_ (20 nm), P25, Micro (200 nm) 0.01, 0.1, and 1 mg/mL	➢ At 1 mg/mL, TiO_2_ aggregates impaired embryo growth. ➢ All TiO_2_ aggregates generated oxidative stress in the absence of solar-simulated radiation. ➢ The nanomaterial P25 was the most phototoxic material among all studied particles.
Brine shrimp *(Artemia franciscana*)	Acute toxicity of TiO_2_, CuO, and ZnO nanoparticles in brine shrimp, Artemia franciscana [22]	TiO_2_ NPs 10, 20, 30, 40, 50, 60, 70, 80, 90, and 100 mg/L	➢ Exhibited moderate toxicity to Artemia larvae in 24 h. ➢ Toxicity of NPs may be the result of the effects of NPs itself, dissolution. Products and NP agglomerates that develop during the experiment. ➢ Indicated that due attention should be paid to the potential effects of NPs on ecotoxicity and environmental health.
Brine shrimp (*Artemia salina*)	Determination of the acute toxic effect of ZnO-TiO_2_ nanoparticles in brine shrimp (Artemia salina) [23]	TiO_2_ (99.0% pure) was obtained in pure anatase.	➢ Artemia showed toxic effects such as enlarged intestines, ocular contraction, pallor of the eye, change in the shape of the eye socket, and deformations in the outer layer.
Brine shrimp *(Artemia salina*)	Effects of aqueous suspensions of titanium dioxide nanoparticles on Artemia salina: Assessment of nanoparticle aggregation, accumulation, and toxicity [24]	TiO_2_ NPs, (99.5% rutile polymorph) 10, 50, and 100 mg/L	➢ Average content in organisms ranged from 0.47 to 3.19 and from 1.29 to 4.43 mg/g in 24 and 96 h, respectively. ➢ Accumulation in adults ranged from 2.30 to 4.19 and from 4.38 to 6.20 mg/g in 24 and 96 h, respectively. ➢ Not toxic to Artemia. ➢ Mortality, such as lipid peroxidation, increased in prolonged exposure to 96 h. ➢ The highest mortality occurred in suspensions of 100 mg/L: 18% for nauplii and 14% for adults (LC50 > 100 mg/L).
Early nauplii of (*Artemia* sp.)	Toxicity of TiO_2_, SiO_2_, ZnO, CuO, Au, and Ag-engineered nanoparticles on hatching and early nauplii of *Artemia* sp. [25]	Nano-TiO_2_ (synthesized) 1, 10, and 100 mg/L	➢ We found a strong correlation between hatching rates and larval mortality in various nanoparticle suspensions. ➢ Found a LC50 of TiO_2_ at 48 h; 18.94 mg/L.
Zebrafish embryos	Adsorption characteristics of nano-TiO_2_ onto zebrafish embryos and its impacts on egg hatching [13]	Nano-TiO_2_ P-25 0, 10, 20, 60, and 120 mg/L	➢ Results showed that the configuration of TiO_2_ aggregates could impact the hatching efficiency of zebrafish embryos. ➢ Adsorption capacity at 0.5–1.0 µg-TiO_2_/#-egg promoted egg hatching, but hatching was inhibited at higher adsorption capacity.
Zebrafish embryos	Efficacy of the hatching event in assessing the embryo toxicity of the nano-sized TiO_2_ particles in zebrafish: A comparison between two different classes of hatching-derived variables [26]	P-25 nTiO_2_ 21 nm 0, 0.01, 10, and 1000 mg/mL	➢ EC10 and EC50 values that caused premature hatching at 58 hpe for nTiO_2_ are 0.073 mg/mL and 107.2 mg/mL, respectively. ➢ The treatment with nTiO_2_ induced significant premature hatching compared to only 6% of the hatched embryos in the control at the same time point.

**Table 2:** Comparison of lethal concentrations using different statistical methods. The results of the Reed and Muench method were used to make the graph estimation and exponential equation. The mean is presented as the standard deviation (*n* = 3).

	LC_20_ (mg/L)	LC_30_ (mg/L)	LC_50_ (mg/L)	LC_80_ (mg/L)
Graph estimation	188.34 ± 19.38	224.34 ± 17.46	269.60 ± 14.88	n.a.
Exponential equation	188.25 ± 20.51	227.46 ± 25.55	288.90 ± 36.44	360.23 ± 52.56
Probit	171.62 ± 20.48	230.50 ± 31.38	345.47 ± 57.23	665.84 ± 148.30
Origin	184.70 ± 21.49	n.a.	293.49 ± 0	470.92 ± 58.50
Average	183.23 ± 7.92	227.43 ± 3.08	301.32 ± 39.43	499.00 ± 154.73

## Data Availability

The datasets generated during the study are available from the corresponding author upon request.

## Supplementary Materials

The following supporting information can be downloaded at https://www.mdpi.com/article/10.3390/nano14040373/s1, Figure S1: Particle agglomeration measure of TiO_2_ P25 NPs.
