# Supplementary material for: Ecotoxicological Effects of TiO2 P25 Nanoparticles Aqueous Suspensions on Zebrafish (Danio rerio) Eleutheroembryos
Source: Nanomaterials (Basel). 2024 Feb 17;14(4):373. doi: 10.3390/nano14040373 (PMC10893039; doi:10.3390/nano14040373)
Supplement: Supplementary file 1 [file nanomaterials-14-00373-s001.zip › nanomaterials-2838148-supplementary.pdf]

Ecotoxicological Effects of TiO_2 P25 Nanoparticles Aqueous Suspensions on Zebrafish (*Danio rerio*) Eleutheroembryos

Melissa I. Ortiz-Román *, Ileska M. Casiano-Muñiz and Felix R. Román-Velázquez *

Department of Chemistry, University of Puerto Rico, Mayaguez Campus,
Mayaguez, PR 00681, USA; ileska.casiano@upr.edu
* Correspondence: melissa.ortiz10@upr.edu (M.I.O.-R.); felixr.roman@upr.edu (F.R.R.-V.)

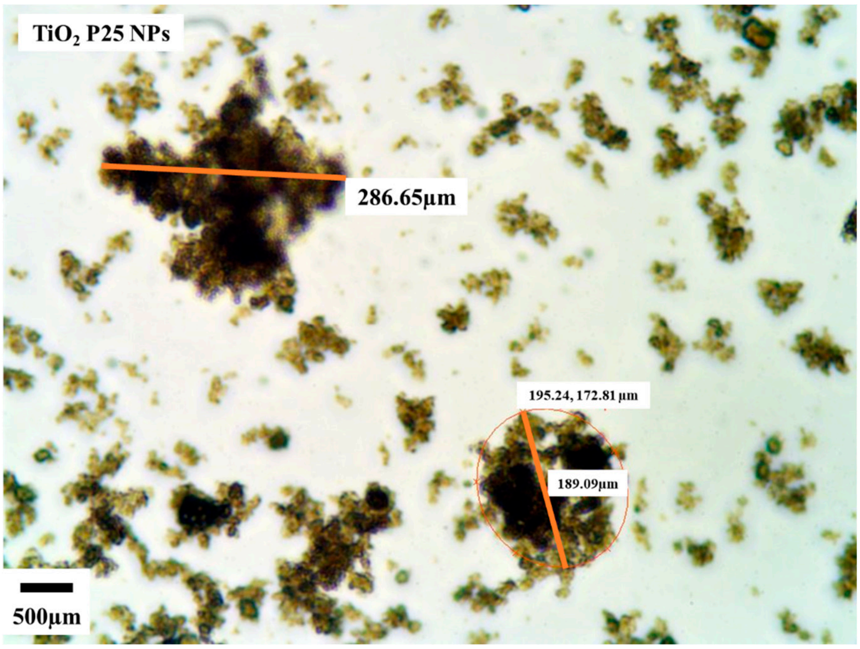

Figure S1. Particle agglomeration measure of TiO_2 P25 NPs
